# The explicit judgment of long durations of several minutes in everyday life: Conscious retrospective memory judgment and the role of affects?

**DOI:** 10.1371/journal.pone.0195397

**Published:** 2018-04-03

**Authors:** Sylvie Droit-Volet, Sophie Monceau, Mickaël Berthon, Panos Trahanias, Michail Maniadakis

**Affiliations:** 1 Université Clermont Auvergne, CNRS, UMR 6324, Clermont-Ferrand, France; 2 Foundation for Research and Technology (FORTH), Hellas, Greece; Duke University, UNITED STATES

## Abstract

In this study, individuals estimated interval times of several minutes (from 2 to 32 minutes) during their everyday lives using a cell phone they kept with them. Their emotional state, the difficulty of the activity performed during this interval, and the attention that it required were also assessed, together with their subjective experience of the passage of time. The results showed that the mean time estimates and their variability increased linearly with increasing interval duration, indicating that the fundamental scalar property of time found for short durations also applies to very long durations of several minutes. In addition, the emotional state and difficulty of the activity were significant predictors of the judgment of long durations. However, the awareness of the passage of time appeared to play a crucial role in the judgment of very long duration in humans. A theory on the emergence of the awareness of the passage of time and how it affects the judgment of interval durations lasting several minutes is therefore discussed.

## Introduction

The human brain is a clock allowing us to accurately measure time [1,2,3]. However, the question is: what is time? Is an internal clock capable of measuring all interval durations independently of the amount of time to be estimated? Most researchers who adhere to the internal clock models accept the idea that the same clock mechanism makes it possible to measure a wide range of interval durations going from a few hundred milliseconds to several minutes [4]. However, they have almost all focused their investigation of time judgment in humans on durations shorter than one minute. It is therefore too early to generalize the timing mechanisms involved in the processing of short durations to that of long durations. One main aim of our experiment was to characterize the judgment of time in the minute range in everyday life.

Very few studies have systematically investigated the timing of interval durations of several minutes in humans using a prospective paradigm in which the participants are explicitly instructed to judge the duration of the upcoming stimulus or event. This is primarily due to methodological obstacles related to the difficulty of finding subjects who are prepared to sit in front of a computer during a long, boring laboratory task, and this several times per day. As a result, the few studies that have examined durations of several minutes have tested interval durations of less than 3–5 minutes [5,6,7,8]. And in cases where longer durations have been used, they have often tested only one or two durations, each of which was presented once (one trial) [9,10,11]. For example, Tobin et al. [11] assigned participants to one of three duration conditions (12, 35 or 58 minutes) and explained to them that they would be interrupted during a video game and asked to judge the length of time they had been playing for. In such a task, it was impossible to measure the variability of time estimates because doing so would have required several trials [12]. This temporal measure is nonetheless essential if we are to test the conformity of minute-duration judgments to the scalar property of time, which is a fundamental property of interval timing. According to this property, the variability of temporal estimates increases (SD) proportionally to the length of the durations, with the result that the coefficient of variation (SD/mean) remains constant when the duration range changes [13]. In the present study, we therefore decided to test a series of interval durations in the minutes range over several trials, but to do so in an ecological condition, i.e., in everyday life, by using a cell phone that the participants kept with them for the day. They therefore went about their daily activities during the intervals.

Intuitively, one might think that it is impossible to track the length of very long durations of several minutes without the help of our watches. The few results for very long durations are inconsistent with this idea because they have shown that it continues to be possible to discriminate durations in the minutes range. Indeed, although studies have only compared 2–3 interval durations, they have found that longer durations are systematically judged longer than shorter ones, for example 24 min is judged longer than 8 min [10], or 35 min longer than 12 min [11]. In addition, in the series of long durations tested, the shortest ones have tended to be overestimated and the longest ones to be underestimated [9,10,14], or overestimated to a lesser extent [11]. Despite the inconsistency in the direction of time estimates, these findings are consistent with Vierordt’s law, with short durations being reported as longer and long durations as shorter than they actually are. This finding cannot therefore result from intrinsic properties of durations in the minutes range since it is also observed for shorter durations of just a few seconds. Instead it reflects the central tendency of judgment described by Hollingworth [15], according to which the judgment of durations is not absolute, but relative to the median of the other durations presented in the series. In the Bayesian framework, this is explained by the influence of temporal context (preceding duration values) on judgment of a given duration [16].

As regards the variability of temporal judgment, only Lewis and Miall [14] have examined the scalar property of time in a study involving a small number of subjects (N = 8) and durations going from 68 s to 16 minutes. In their study, the coefficient of variation appeared to be quite large (CV = 0.35). However, and more critically, they observed a violation of the scalar property of time, with a non-constant CV across durations. However, as the authors explained, “it seems prudent to treat this initial finding with caution and even some suspicion” (p. 1900). This finding probably related to the task used (temporal reproduction), as well as to the secondary task used for the presentation and reproduction phases. In addition, the discontinuity in the CV, which might have indicated some changes in the timing mechanisms at approximately 1.5 s [17], was not found in their results in the critical intervals at about 1.5 s or 3–4 s.

Whatever the results, it is obvious that different mechanisms exist for different ranges of interval durations. We can presume that the estimation of very long durations cannot result from a number of counts (pulses) accumulated in a “timer” in an uninterrupted and regular way over a period of several minutes. The number of pulses accumulated is also dependent on the way attention is paid to time [18], and it is unlikely that the subjects are able to maintain the same level of attention over a very long period of time [11]. In addition, at the level of seconds, the mechanisms involved in the processing of durations are already different from those involved in the processing of shorter durations of the order of milliseconds, with more attention and working memory processes being devoted to the former [19,20]. As discussed later, although the mechanisms involved in the processing of long durations in everyday personal experiences have not as yet been identified, we can assume that additional processes related to long-term memory (episodic memory) are involved in the measurement of these durations.

Recently, Droit-Volet and her collaborators examined the relation between the judgment of durations and the subjective experience of the passage of time [21,22]. The passage of time judgment (PoTJ) is the awareness of the flow of time and its variation in comparison to the conventional representation (knowledge) of the constant rhythm of an external clock [23,24]. It results from a reflexive, moment-by-moment introspection on the self in time (self-time), i.e. the consciousness of the relationship between the pace of self-time and time in the external world. They observed that the judgment of durations is not related to PoTJ in the short duration ranges: 350 ms *vs*. 1650 ms [21], and 2.8 s *vs*. 33 s [22]. However, a link between these two forms of time judgment appears for durations in the range of minutes (2, 4, 6, 8 min) [22]. As we discuss later, this suggests that the judgment of long durations and the PoTJ would share some of the high-order cognitive mechanisms involved in the recovery of long durations in long-term memory, i.e. the retrospective memory retrieval of the non-temporal content of the temporal interval. Therefore, the PoTJ during the long interval to be estimated, as well as the contextual content of this interval, were also assessed in our study after each estimate of interval duration. Some studies have shown that the PoTJ depends on the level of emotion and the activity performed at the moment of the PoTJ. Participants do indeed perceive time as passing faster when they feel more excited and aroused. Although it has been observed less consistently across studies, the difficulty of the activity and the attention that it captures are also significant predictors of PoTJ. Consequently, to further examine the determinants of judgments of very long durations, we assessed the PoTJ, the affective states felt and the activity (difficulty, attention) performed during the interval duration. In our study, which was designed to examine the prospective time judgment of very long durations, the participants were therefore given a verbal estimation task in which they had to judge several interval durations, from 2 to 32 min, during their everyday life and also had to assess the feeling the passage of time, the emotion felt and the activity performed during these interval durations.

## Method

### Participants

The sample consisted of 60 participants (Mean Age = 28.44, SD = 6.46; 40 women and 20 men). The results for one participant were excluded due to a technical problem with the cell phone. A consent form describing the constraints necessarily involved in the experiment was signed by each individual before his or her participation in this study. This study was conducted in accordance with the 1964 Helsinki declaration and was approved by the Sud-Est VI Statutory Ethics Committee of France. The participants received 20 euros for their participation.

### Material

The participants were given a Motorola G Androit Jelly Bean smartphone, with a program written by CATech (http://lapsco.univ-bpclermont.fr/catech) at Clermont Auvergne University. This program managed all the events: alerts, temporal tasks, questions, recording of responses. The participants gave their responses by pressing on the touch screen of the cell phone. The stimulus at the onset and the offset of the interval duration was a sound (LA, 440 Hz).

### Procedure

The participants were randomly assigned to one of the two duration conditions: 2/8-min (30 participants) and 8/32-min (29 participants). In each duration condition, there were 7 target interval durations to be estimated. The interval durations were 2, 3, 4, 5, 6, 7, and 8 min in the 2/8-min condition, and 8, 12, 16, 20, 24, 28 and 32 min in the 8/32-min condition. Each target duration was presented 3 times, together with 5 other durations randomly chosen between 1 and 16 for the 2/8-min condition, and between 4 and 64 minutes for the 8/32-min condition. This represented a total of 26 trials. The 26 trials were presented randomly during the day, the inter-trial interval being between 10 and 17 min (M = 13.5, *SD* = 2.3). The procedure was therefore similar in the two duration conditions, except for the length of the interval durations to be estimated and the consequence of this on the duration of the session, with the trials ranging from 7.00 a.m. to 7.30 p.m. for the 2/8-min condition and from 7.00 a.m. to 11.55 p.m. for the 8/32-min condition.

For each trial, an alert given by the cell phone indicated that a new trial was to be performed. The participant touched the phone screen to stop this alert. The word "ready" then appeared and the participant again touched the phone screen to trigger the trial. The participants therefore heard two sounds separated by the interval duration to be judged. The length of the first sound was 2 s and the second stopped when the participants touched the cell phone in order to respond. The participants then gave their estimate of the interval duration by pressing on the touch screen of the cell phone and received no feedback.

After each verbal estimate, the participants answered 7 questions on their cell phones. The first (1) was the question on the speed of the passage of time compared to the notion of a constant clock rhythm (PoTJ): “how is time passing for you between the 2 sounds, compared to the time of the clock, from "much slower" to "much faster?”. The participant responded to this question on a 7-point scale going from "much slower" to "much faster". The next 4 questions related to the affect they had experienced during the interval between the two sounds: (2) “excited/stimulated” (Arousal), (3) “relaxed/calm” (Relaxation), (4) “happy” (Happiness), and (5) “sad” (Sadness). The last two questions then related to the activity performed between the two sounds: (6) if it was difficult and (7) if it had captured all their attention. For the affective and the activity questions, the participants responded on a 7-point scale from “not at all” to “very much”.

The day before the experimental session, the participants were instructed that they would have to judge the duration between two sounds and two demonstration trials were administered with an interval of a few seconds. They were told only that the durations would be between 1 and 16 minutes for the 2/8-min condition and between 4 and 64 min for the 8/32-min condition. The participants activated their cell phones on the morning of the day of the test session and kept their cell phone with them during the whole day. They were required to stay at home during this specific day. They were also instructed to remove all clocks (watches, cell phone clock, clock) because looking at a clock would have biased the scientific results. They were only allowed to consult a watch or their cell phone under exceptional circumstances between two trials if they judged this to be absolutely necessary. The participants, who were paid, agreed to comply with these experimental constraints before participating in this study. They were also instructed not to count, even though this would have been impossible for the very long durations. As indicated by the results, the variability of temporal estimates did not remain constant but increased with the duration length, as expected due to the lack of a counting strategy [25].

## Results

### Temporal estimates of long durations

#### Mean duration

**Fig 1:**
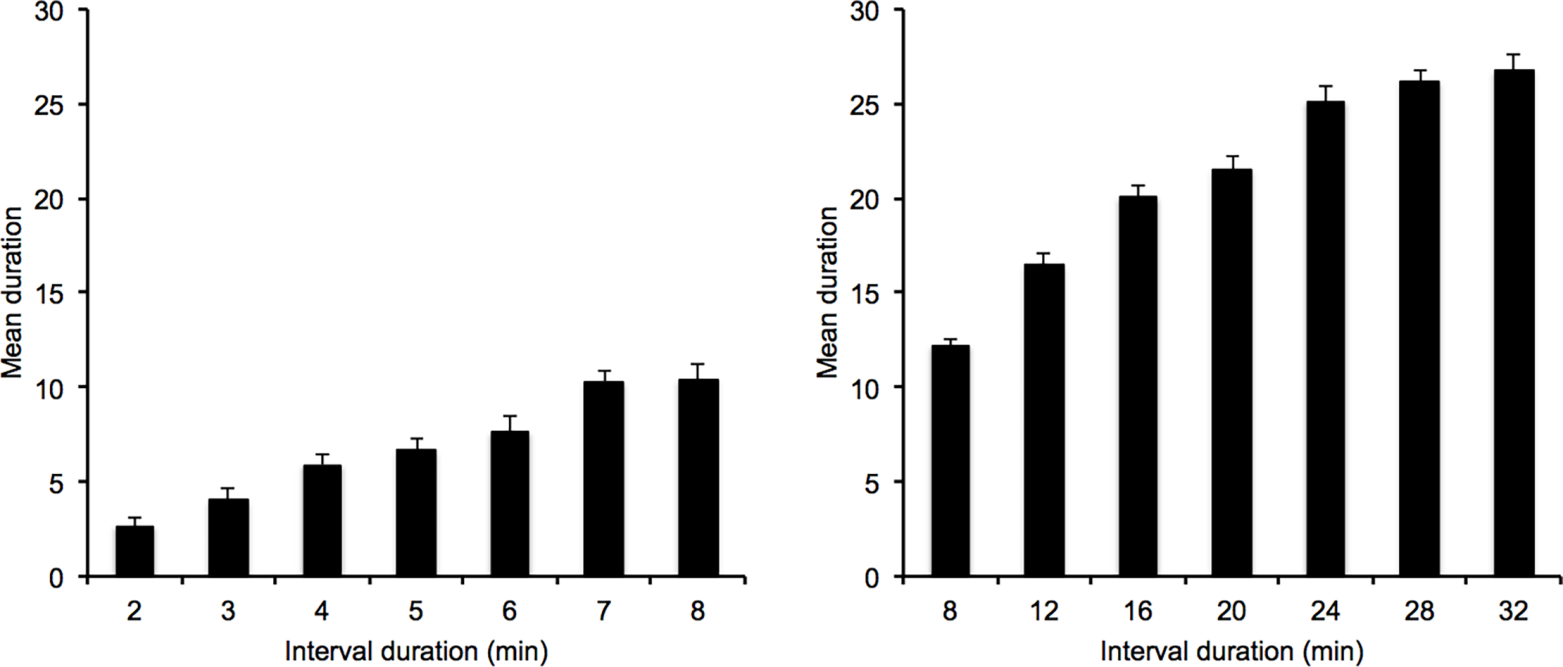
Mean duration. Mean estimated duration plotted against interval duration in the 2/8 and the 8/32-min condition.

The mean estimated duration was calculated for the target interval durations in the 2/8-min and the 8/32-min condition (Fig 1). An initial statistical analysis was conducted on this temporal measure that did not find any effect of the sex factor. This factor was thus excluded from the subsequent analyses. An ANOVA was performed on the mean estimated duration using IBM SPSS, with the duration condition as between-subjects factor and the interval durations as within-subjects factor (7 interval durations). This ANOVA showed significant main effects of interval duration, *F*(6, 342) = 99.62, *p* = .0001, *η*^*2*^_*p*_ = .64, and duration condition, *F*(1, 57) = 787.27, *p* = .0001, *η*^*2*^_*p*_ = .93, as well an interval duration x duration condition interaction, *F*(6, 342) = 10.50, *p* = .0001, *η*^*2*^_*p*_ = .16. For each duration condition taken separately, there was a linear effect of interval duration (2/8-min, *F*(1, 29) = 372.12, *p* = .0001, *η*^*2*^_*p*_ = .93; 8/32 min, *F*(1, 28) = 243.80, *p* = .0001, *η*^*2*^_*p*_ = .90), indicating an increase in the mean temporal estimates as the duration value increased. In other words, time discrimination was maintained for very long durations of several minutes. However, the post-hoc comparisons using the Bonferroni test in the 8/32-min condition revealed that the mean of the estimated durations did not differ between the interval durations of more than 24 min (24 *vs*. 28, 24 *vs*. 32, 28 *vs*. 32, *p* > .05), although these durations differed from the other shorter interval durations. Similarly, in the 2/8-min condition, there was no difference between the temporal estimates for the two longest interval durations (7 *vs*. 8 s, *p* > .05).

#### Standardized error

**Fig 2:**
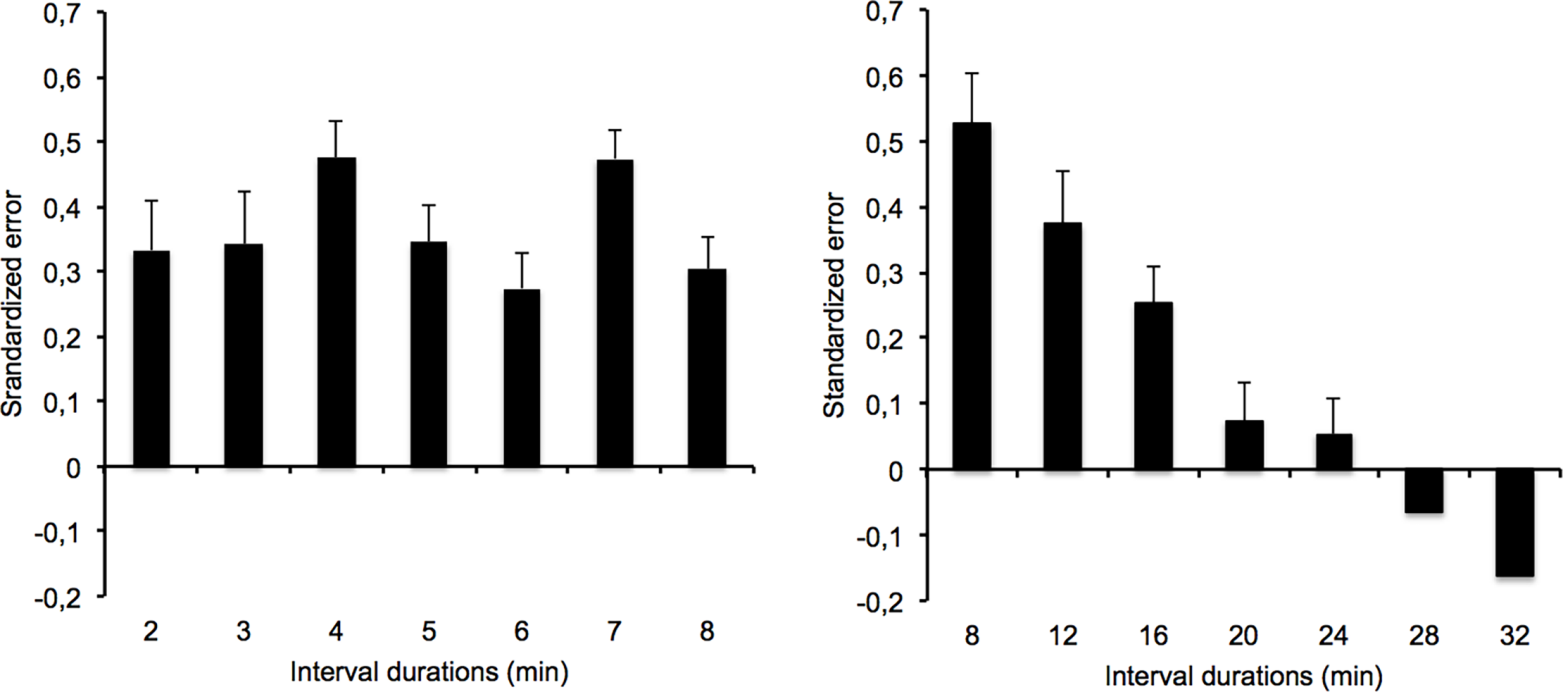
Standardized error. Standardized error plotted against interval duration in the 2/8 and the 8/32-min condition.

To examine the size of the deviation of the time estimates from the target durations, we calculated an index of standardized error that was the difference between the time estimates and the target duration, divided by the target duration. The ANOVA conducted on this new measure showed a significant main effect of the duration condition, *F*(1, 57) = 15.44, *p* = .0001, *η*^*2*^_*p*_ = .23, indicating that the overestimation of long durations was greater in the 2/8-min condition than in the 8/32-min condition (0.36 vs. 0.15). However, there was also a significant main effect of the interval duration, *F*(6, 342) = 12.58, *p* = .0001, *η*^*2*^_*p*_ = .18, and a significant interaction between the interval duration and the duration condition, *F*(6, 342) = 12.58, *p* = .0001, *η*^*2*^_*p*_ = .18. The ANOVA conducted on each duration condition taken separately showed no significance of the interval duration, *F*(6, 174) = 1.97, *p* = .072, in the 2/8-min condition, while this factor was significant in the 8/32-min condition, *F*(6, 168) = 29.65, *p* = .0001, *η*^*2*^_*p*_ = .51. As illustrated in Fig 2, in the 2/8-min condition, the interval durations were all overestimated, with the time estimates being significantly different from zero (*p* < .05 for all *t* tests) and the same magnitude of overestimation occurring across the different interval durations. By contrast, in the 8/32-min duration condition (8/32 min), the time estimates for the shortest interval durations were also different from zero and overestimated (8 min, *t*(28) = 8.08; 12 min, *t*(28) = 5.62; 16 min, *t*(28) = 5.18, all *p* < .05), while those for the longest durations were underestimated (28 min, *t*(28) = -2.52; 32 min, *t*(28) = -4.80, *p* < .05), with a central time judgment at 20 and 24 min (*t*(28) = 1.55, *t*(28) = 1.17, respectively, both *p* > .05).

#### Standard deviation

**Fig 3:**
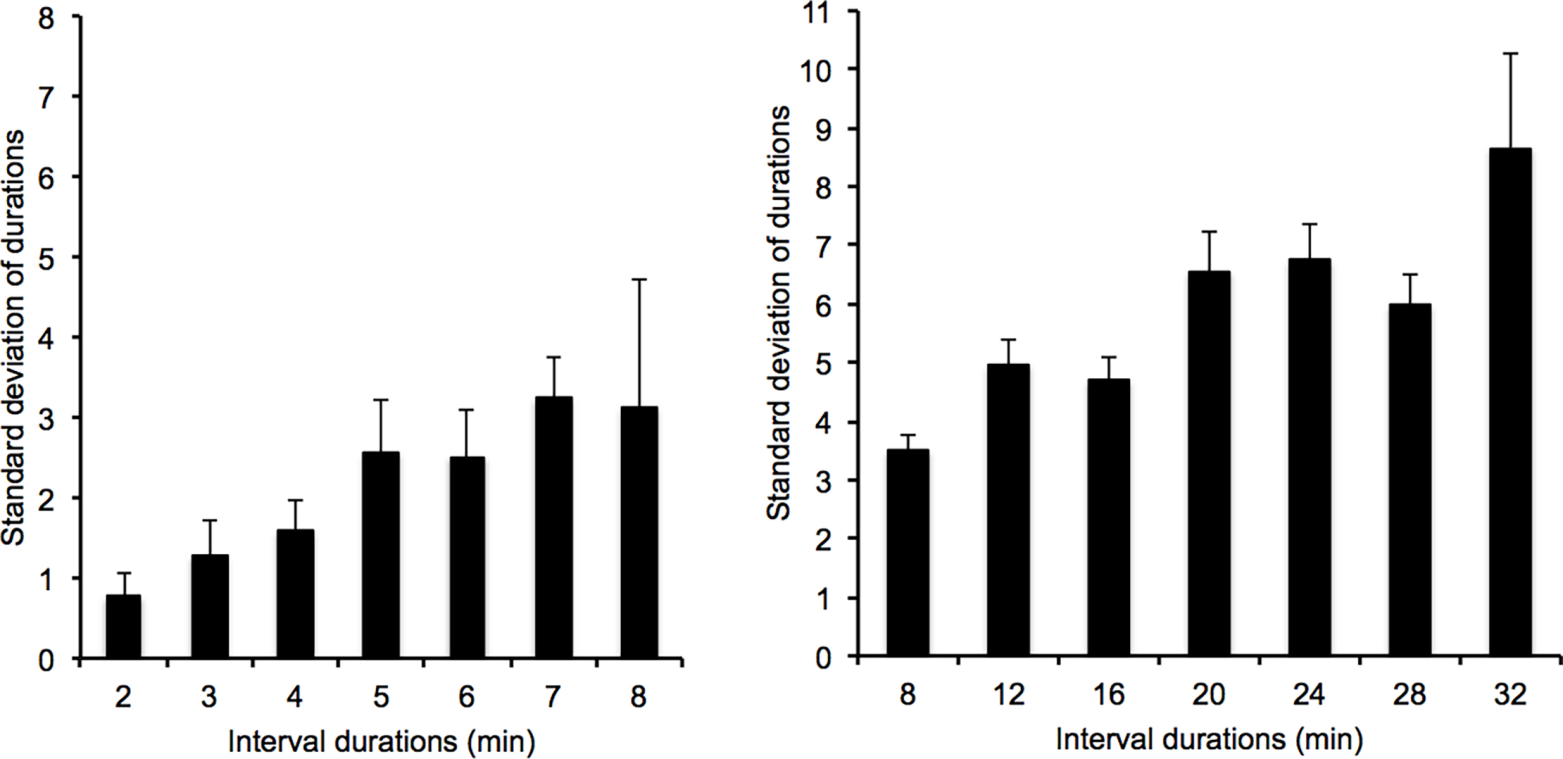
Variability of time estimates. Standard deviation of estimates plotted against interval duration in the 2/8 and the 8/32-min condition.

Another ANOVA was performed on the variability of time estimates (SD) (Fig 3). This analysis showed a significant main effect of the interval duration, *F*(6, 342) = 5.44, *p* = .0001, *η*^*2*^_*p*_ = .09, and of the duration condition, *F*(1, 57) = 99.93, *p* = .0001, *η*^*2*^_*p*_ = .64, while the interval duration x duration condition interaction did not reach significance, *F*(6, 342) = 0.84, *p* = .54. Consequently, the variability of time estimates was larger in the long than in the shorter duration condition (2.15 vs. 4.21), and it linearly increased with the length of the interval durations whatever the duration condition, *F*(1, 55) = 17.37, *p* = .0001, *η*^*2*^_*p*_ = 0.23. In accordance with the scalar property of time, when the SD was divided by the target duration (coefficient of variation) (Table 1), these effects were no longer significant. This was true of the interval duration, *F*(6, 342) = 1.61, *p* = .14, the duration condition, *F*(1, 57) = 1.08, *p* = .30, and the interaction between them, *F*(6, 342) = 0.34, *p* = .91. In each duration condition considered separately, we did indeed find a linear effect of interval duration on SD for both the 2/8-min condition, *F*(1, 29) = 71.79, *p* = .0001, *η*^*2*^_*p*_ = .71, and the 8/32-min condition, *F*(1, 28) = 6.72, *p* = .015, *η*^*2*^_*p*_ = .19, with no effect of interval duration being observed on the coefficient of variation, *F*(6, 174) = 1.26, *p* = .28, *F*(6, 168) = 0.83, *p* = .55, respectively.

**Table 1:** Temporal measures of judgment of interval durations in the 2/8-min and the 8/32-min duration condition.

	Mean duration		Standardized errors		Standard deviation		Coef. of variation	
	Mean	SE	Mean	SE	Mean	SE	Mean	SE
***2/8-min condition***								
**2 min**	2.67	0.38	0.33	0.08	0.77	0.28	0.30	0.03
**3 min**	4.03	0.58	0.34	0.08	1.27	0.44	0.30	0.04
**4 min**	5.90	0.57	0.48	0.06	1.58	0.38	0.25	0.03
**5 min**	6.63	0.71	0.35	0.06	2.56	0.67	0.37	0.04
**6 min**	7.64	0.79	0.27	0.05	2.51	0.59	0.32	0.04
**7 min**	10.32	0.57	0.47	0.04	3.24	0.51	0.31	0.03
**8 min**	10.40	0.83	0.30	0.05	3.12	1.58	0.31	0.06
***8/32-min condition***								
**8 min**	12.21	0.38	0.53	0.08	3.50	0.28	0.29	0.03
**12 min**	16.48	0.59	0.37	0.08	4.97	0.45	0.31	0.04
**16 min**	20.05	0.58	0.25	0.06	4.70	0.39	0.24	0.03
**20 min**	21.51	0.72	0.08	0.06	6.56	0.68	0.34	0.04
**24 min**	25.15	0.80	0.05	0.05	6.75	0.60	0.30	0.04
**28 min**	26.19	0.58	-0.07	0.04	5.98	0.52	0.23	0.03
**32 min**	26.79	0.84	-0.16	0.05	8.66	1.61	0.33	0.06

In addition, as the same interval duration of 8 min was used in the two duration conditions, we conducted a one-way ANOVA for each temporal measure for this specific interval duration. This showed no difference in the temporal variability between the duration conditions for the SD, *F*(1, 57) = 0.59, *p* = .44, or the coefficient of variation, *F*(1, 57) = 0.34, *p* = .56. The 8-min duration was nevertheless judged longer in the 8/32-min (M = 12.20, SE = 0.52) than in the 2/8-mn condition (M = 10.40, SE = 0.51), *F*(1, 57) = 6.15, *p* = .02, *η*^*2*^_*p*_ = .097, and a higher standardized error was observed, *F*(1, 57) = 5.93, *p* = .02, *η*^*2*^_*p*_ = .094, thus confirming the influence of temporal context [16,26].

### The predictors of time estimates of long durations

On each trial, the participants reported their judgment of the passage of time during the interval duration (inter-sound interval), their affective state and the level of difficulty of the activity performed during this interval, and the extent to which it captured their attention. We analyzed which factor might potentially be a significant predictor of their time estimates (mean estimated durations) by using multilevel modeling (MLM) in the SPSS software (for the same procedure with similar measures and a description of this model, see [21,22,23,24]). Since the temporal measure (mean estimated durations) changed as a function of the duration condition, we decided to conduct these analyses for each duration condition taken separately.

For both the 2/8-min condition (Table 2) and the 8/32-min condition (Table 3), the best predictor of the verbal estimation of durations lasting several minutes was the passage of time judgment (PoTJ) (*p* = .0001). The interval duration was therefore judged shorter when time was experienced as passing faster, and increased when it seemed to pass slower. The other factor that significantly predicted the estimation of interval durations of several minutes was the affective state, i.e., the level of arousal (excitement/stimulation) felt during the interval (2/8-min, *p* = .04; 8/32-min, *p* = .004). The higher the arousal level was, the shorter the interval duration was judged to be. In other words, the interval duration was judged longer when the self-reported level of arousal decreased. Our results suggest that the estimation of long durations of several minutes was not linked to other factors, such as those related to the affective valence (happiness, sadness) or the level of attention devoted to the current activity (difficulty, attention). However, when all duration conditions were included in the same statistical analysis (Table 4), these factors also reached significance (*p* < .05), except for the attention paid to the current activity, which just failed to reach significance (*p* = .09). Consequently, when a larger number of trials was considered, the estimated durations became shorter as the level of task-difficulty increased. As far as affective valence is concerned, the time estimates lengthened with the level of happiness, and shortened with that of sadness. The analyses for the standardized error are not reported, but found that the PoTJ was the main significant predictor of inter-individual differences in the magnitude of temporal judgment errors (*Estimate* = -0.055071, *SE* = 0.0114, *t* = -4.84, *p* = .0001).

**Table 2:** Potential predictors of mean estimated duration for the 2/8-min condition. The predictor is shown along with its associated estimate (coefficient), the confidence interval, the standard errors, *t*-score and *p* value.

Predictor	Estimate	Confid. Int.	SE	*t* value	*p* value
**PoTJ**	-60866.39	[-74016.37; -47716.43]	6694.06	9.09	0.0001
**Aroused**	-16210.76	[-31926.67; -494.86]	7996.11	2.03	0.043
**Relaxed**	1000.16	[-13774.79; 15775.11]	7513.14	0.13	0.89
**Happiness**	2680.89	[-15353.76; 20715.545]	9158.53	0.29	0.77
**Sadness**	-19076.73	[-36880.41; -1273.05]	8985.33	2.12	0.036
**Activity difficulty**	5651.24	[-4534.06; 15836.54]	5176.67	1.09	0.28
**Activity attention**	2174.45	[-8072.07; 12420.96]	5212.19	0.42	0.68

**Table 3:** Potential predictors of mean estimated duration for the 8/32-min condition. The predictor is shown along with its associated estimate (coefficient), the confidence interval, the standard errors, *t*-score and *p* value.

Predictor	Estimate	Confid. Int.	SE	*t* value	*p* value
**PoTJ**	-105353.6	[-136933.08; -73774.12]	16076.95	6.55	0.0001
**Aroused**	-44141.49	[-74365.61; -13917.38]	15373.83	2.87	0.004
**Relaxed**	21557.46	[-7213.83; 50328.74]	14643.7	1.47	0.14
**Happiness**	-31130.14	[-68638; 6378.08]	18922.08	1.65	0.10
**Sadness**	-9051.08	[-49519.36; 31417.21]	20401.31	0.44	0.66
**Activity difficulty**	-11823.36	[-34247.66; 10600.94]	11404.21	1.04	0.30
**Activity attention**	-6810.46	[-30674.50; 17053.57]	12135.52	0.56	0.58

**Table 4:** Potential predictors of mean estimated duration for the two conditions together (2/8-min and 8/32-min condition). The predictor is shown along with its associated estimate (coefficient), the confidence interval, the standard errors, *t*-score and *p* value.

Predictor	Estimate	Confid. Int.	SE	*t* value	*p* value
**PoTJ**	-97486.18	[-121377.60; -73594.76]	12173.33	8.01	0.0001
**Aroused**	-64691.16	[-89078.59; -40303.73]	12430.01	5.20	0.0001
**Relaxed**	44183.64	[21276.92; 67090.37]	11675.31	3.78	0.0001
**Happiness**	28534.95	[1618.25; 55451.65]	13719.15	2.08	0.038
**Sadness**	-27391.34	[-54030.32; -752.37]	13577.60	2.02	0.044
**Activity difficulty**	-21001.14	[-37496.07; -4506.21]	8407.29	2.50	0.013
**Activity attention**	-14985.42	[-32346.55: 2375.70]	8848.79	1.69	0.091

While the verbal estimation of durations in the minute range was significantly linked to the subjective experience of the passage of time, the passage of time judgment was significantly related to affective states felt as well as to the activity performed during the interval duration (Tables 5 and 6). The PoTJ is thus highly dependent on the non-temporal context experienced during the interval. Indeed, the MLM analyses conducted on the PoTJ in the two duration conditions demonstrated that the emotional states (arousal, relaxation, happiness, sadness) and the current activity (difficulty, attention) were significant predictors of PoTJ. These results replicated those found in previous studies on the PoTJ judgment [21,22,23,24]. Indeed, as Tables 5 and 6 show, the participants perceived time as passing faster when they felt more aroused and happier (*p* < .001). Time was also experienced as passing faster when the activity undertaken during the temporal interval was more difficult and captured more attention (*p* < .01). Conversely, the passage of time seemed to slow down the more relaxed or sad the participants felt.

**Table 5:** Potential predictors of passage of time judgment for the 2/8-min condition. The predictor is shown along with its associated estimate (coefficient), the confidence interval, the standard errors, *t*-score and *p* value.

Predictor	Estimate	Confid. Int.	SE	*t* value	*p* value
**Aroused**	0.199	[0.107; 0.292]	0.047	4.29	0.0001
**Relaxed**	-0.112	[-0.198; -0.027]	0.043	2.57	0.01
**Happiness**	0.155	[0.044; 0.266]	0.564	2.76	0.007
**Sadness**	-0.116	[-0.234; 0.001]	0.059	1.94	0.05
**Activity difficulty**	0.086	[0.026; 0.146]	0.030	2.82	0.005
**Activity attention**	0.130	[0.068; 0.192]	0.031	4.16	0.0001

**Table 6:** Potential predictors of passage of time judgment for the 8/32-min condition. The predictor is shown along with its associated estimate (coefficient), the confidence interval, the standard errors, *t*-score and *p* value.

Predictor	Estimate	Confid. Int.	SE	*t* value	*p* value
**Aroused**	0.234	[0.157; 0.313]	0.040	5.94	0.0001
**Relaxed**	-0.104	[-0.181; -0.028]	0.389	2.68	0.008
**Happiness**	0.148	[0.047; 0.248]	0.051	2.90	0.004
**Sadness**	-0.597	[-0.184; 0.065]	0.060	0.99	0.33
**Activity difficulty**	0.101	[0.030; 0.172]	0.036	2.84	0.006
**Activity attention**	0.084	[0.018; 0.149]	0.033	2.52	0.01

## Discussion

Our study on the verbal judgment of times lasting several minutes revealed humans’ ability to judge very long durations. Indeed, our results showed that the mean time estimate increased linearly with increasing interval duration, although it did not match the real duration, as indicated by the overestimation of most of the interval durations, and the underestimation of durations longer than 24 minutes. In addition, the variability of temporal estimates increased linearly with the length of the durations, with the coefficient of variation remaining constant as the durations to be judged changed. Overall, these results highlight the fact that the fundamental scalar property of time observed for short durations (< 1 min) is also found for long durations of several minutes. This suggests that a common process, probably an internal clock-like system, is involved in the timing of different time scales going from a few hundred milliseconds to several minutes or, as we speculate in our theoretical section below, in the timing of a series of shorter interval/event durations (< 30 s) that are stored in long-term memory and fill temporal spans of several minutes. However, several original findings in our study suggest that processes other than the clock system also contribute to the time judgment of very long durations, at least in the case of explicit temporal judgments made by humans.

**Fig 4:**
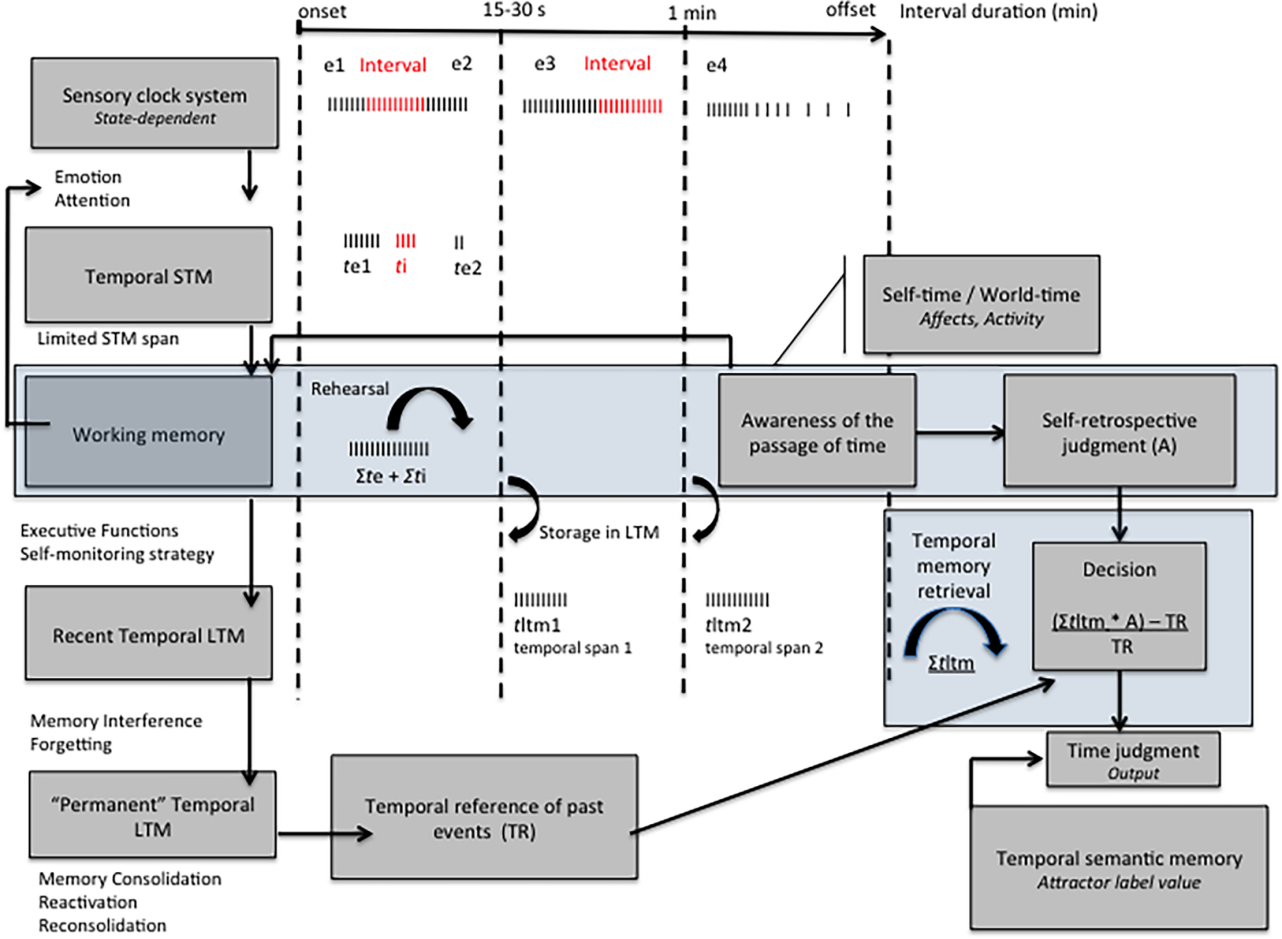
Theory of explicit time judgment of very long durations.

As noted in the introduction, there are many reasons why only a few studies have examined the timing of durations lasting several minutes. One of these is that some researchers believe that the clock system could work online for a long period of time in a sort of “run-mode” [27,28,29]. However, temporal information processing depends on short-term and working memory systems with limited capacities [30,31,32]. Short-term memory holds a limited amount of information for a limited time of about 15 to 30 seconds. The rehearsal process can nevertheless increase the length of time that temporal information is retained in working memory. However, it has been demonstrated that maintaining temporal information in working memory produces shortening effects on time judgments (loss of pulses) [33,34], rather than lengthening effects, such as those observed in our study, at least in the case of intervals shorter than 24 min. Consequently, we can assume that the timing of intervals covering several minutes and composed of different events might result from the sum of durations associated with the events (*t*e) (shorter temporal spans) and with the inter-event intervals (*t*i) that fill the long period of time. Therefore, as illustrated in our theory of explicit time judgment of very long durations (Fig 4), the total value of a long interval of time would depend on the temporal content of this interval, i.e., the sum of these local durations (*t*e) and inter-event intervals (*t*i), successively stored in long-term memory (∑*t*ltm) (Fig 4). Based on the internal clock models, we can assume that these local times, *t*e and *t*i, directly depend on the functioning of the clock system and its fluctuation as a function of the context experienced during the interval. Individual state-dependent time processing has been widely demonstrated, namely in studies on emotion showing, for example, how the clock speeds up in frightening conditions and lengthens perceived time [35,36,37].

However, our findings on the explicit judgment of very long durations suggest that the final temporal judgment cannot be reduced to the accumulation of a total quantity of temporal units (pulses) or to the sum of event-specific temporal quantities stored in long-term memory. Indeed, they indicated that the awareness of the passage of times (PoTJ) was a significant predictor of the judgment of long durations from 2 to 32 minutes. Indeed, the length of time estimates increased with the awareness of the slowing down of passage of time. Previous studies have shown no significant link between the judgment of durations and the PoTJ for durations shorter than 1–2 min [21,22]. Participants are always able to judge the speed of the passage of time when they are explicitly asked to do so, even for very short durations. However, based on our results showing that a significant link between the PoTJ and the time judgment only occurred from 1–2 min, we can assume that participants do feel the passage of time—they become spontaneously aware of the passage of time—when they are confronted with its extension, i.e. when the duration extends beyond 1–2 minutes (Fig 4). As Fraisse stated, the sensation of duration appears in consciousness when time expands and becomes burdensome for the activity [38]. We can therefore assume that when the awareness of the passage of time emerges (this sensation of lasting), it begins to affect time judgments (Fig 4). This can partly explain our data showing that all the durations were overestimated compared to the target durations, at least up to a cut-off point at about 25 minutes. Awareness of the passage of time may thus increase the saliency of time and make us attentive to time (self-monitoring strategy), with the result that it appears to lengthen. However, after 20–25 min, an underestimation of time seems to appear, suggesting that other mechanisms occur once the temporal scale shifts to this length.

The role of awareness of the passage time in prospective duration judgments must be further investigated. Nevertheless, we can try to discuss how awareness of the passage of time and its variations affects time judgment. In our theory of the explicit judgment of very long durations (Fig 4), we assume that its action mainly occurs at the level of the decisional processes, and of the memory process responsible for the retrospective judgment of the long temporal span. At this point, a link may be established between the judgment of very long durations and time judgments made within a retrospective paradigm in which the participants do not know in advance that they will have to judge time [8,39,40,41]. The different theories of retrospective time judgment consider that this type of temporal judgment is based on non-temporal information remembered in memory rather than on temporal information encoded in memory: e.g., the amount of information [42], the number of contextual changes [8], or the memory load [43,44]. However, in our results on the prospective judgment of very long durations, we did not find any such simple relationship between the non-temporal information and time estimates. For instance, when the task difficulty increased, the time estimates did not increase but decreased. This is consistent with the studies showing that different processes underpin prospective and retrospective time judgment. For example, it has been shown that some task variables (easy vs. difficult) influence prospective time judgment but not retrospective time judgment [8,45], while other variables influence the former and not the latter. Miller, Hick and Willette [42] also showed that the rehearsal of information increased time estimates in a prospective paradigm, whereas these estimates fell in a retrospective paradigm.

Therefore, the prospective judgment of durations lasting several minutes cannot be reduced to a retrospective-type judgment—based on non-temporal information–that would replace an underlying clock-based representation of time. In other words, this means that the judgment of very long durations is not a simple translation of the awareness of the passage of time based on non-temporal content of interval. Instead, we suppose that there is an interaction between different mechanisms, with the result that the retrospective awareness of the passage of time would act as a “multiplicative parameter” (*A*) that alters output judgment. In this way, the reported duration would increase or decrease as a function of the awareness of a speeding-up (*A* < 1.0) or a slowing-down of the passage of time (*A* > 1.0). This assumption must be examined in experimental studies that allow us to clearly establish a causal link between the PoTJ and the judgment of long durations. However, as explained below, our results suggest that what determines the awareness of the passage of time (*A*) would not be temporal information *per se*, but the characteristics of what is experienced during the interval as self-reported by the participants (affects, activity), i.e. non-temporal information that is also used in the retrospective judgment of time.

The awareness of the passage of time would therefore influence the judgment of durations lasting several minutes. In our study, the significant predictors of PoTJ were the emotion or the difficulty of the activity performed during the interval and the attention that it required. These findings replicated those found by the other studies on the PoTJ [21,22,24]. The participants thus feel that time accelerates when they feel more aroused and happier, and, conversely, that it slows down when they feel more relaxed and sadder. The passage of time is also consciously perceived as faster when the task becomes more difficult. Our data nevertheless suggested that the best, or the most consistent, predictor of PoTJ relates to the emotional state felt during the interval rather than to the activity performed. This emphasizes the importance of the participants' introspective analysis of their internal state for the feeling of the passage of time and its variations (see discussion on the embodiment of time [46]). However, the inconsistency of the activity-related effects on PoTJ is likely to be due to the inter-subject variability in everyday activities. Our results did indeed indicate that this factor reached significance when more subjects were entered in the statistical analyses (Table 4).

As regards the emotional state experienced by the participants, we found an original result suggesting that the relationship between time and emotion changes as a function of the temporal scale. Numerous studies conducted in the milliseconds and seconds range have shown a lengthening of time in conditions of highly arousing emotions [47,48,49]. In contrast, in our study with long durations, we observed not a lengthening but, instead, a shortening of perceived time as the arousal level increased. The higher the arousal level was, the shorter the interval duration was judged to be. The direction of the time judgment (lengthening vs. shortening) therefore seems to reverse between the short and the long duration range. Consequently, the effect of emotion on time cannot be generalized to all temporal scales.

This result can in part be explained by the dynamic of the emotional state. The emotional level induced by an emotional stimulus is often intense at first, but it quickly decreases and leads, in some cases, to a general mood, i.e., an emotional state that is more permanent but less intense [50,51]. For short durations (< 2–8 s), when the emotional reaction is still intense in response to a high-arousal stimulus, this automatically affects the rate of the clock system and consequently also produces time distortions. However, for longer durations (> 1 min) with the same high-arousal stimulus, the emotional reaction decreases in intensity. The clock-based effect on time judgment then disappears to leave space for other mechanisms. In line with this idea, studies on the judgment of the presentation duration of emotional stimuli have shown a rapid decline of emotional effect on time perception after 2 s [52,53], although the emotional effect may be extended (2–8 s) in some particularly frightening situations, for example when the participants expect to receive an electric shock [54]. Therefore, in comparison to the initial dilation of time, a shortening effect appears due to the fall in intensity of the emotional reaction over time. The exact nature of the mechanisms involved in the shortening effect observed in our study must be better defined. However, the relationship between mean estimates and the self-awareness of an acceleration of time with increasing arousal level suggests that these mechanisms consist in attentional executive control processes (endogenous top-down process), through which the subject consciously decides to keep track of what is happening. Intentional monitoring of the content of the temporal interval would contribute to our awareness of variations in the speed of the passage of time that would act as a multiplicative effect with the underlying representation of time. In addition, when the subject paid attention to what is happening (non-temporal information), the time estimates shortened, consistently with the predictions of attentional models of time [18].

Among the additional factors that underpin the judgment of very long durations, we must also consider the role of knowledge of durations of past events stored in long-term memory (temporal reference of past event, *TR*). In our theory, we assume that, at the decisional level, the final response could be based on a decisional rule similar to that used in scalar expectancy theory (SET) [27,55], i.e. the difference between (Σ*t*mlt * A) and *TR*, divided by *TR* (Fig 4). The participant’s verbal response would therefore be based on the comparison between the durations of the current and past events.

In addition, in our study, we employed a verbal estimation task in which the participants used a symbolic representation of time in the form of minutes and seconds (verbal output value). Verbal estimation tasks are often used in studies on time perception, but there is no existing model of how participants perform this task, except for the recent “attractor model” proposed by Wearden [56]. However, this model takes account of short values (ms) and the violation of the scalar property (fall in CV with increasing duration), which were not verified in our experiment. As explained by Wearden [56], this “model operates according to simple principles” (p. 22). The verbal output is not a direct translation of the underlying time representation. The temporal label would play a role of attractor as a function of the distance between the time representation, *t*, and the verbal output. Consequently, some verbal outputs would have more power to “distort expression of the property of underlying time representation” than others. The judgment of an interval duration of 22 minutes is, for example, more often equal to 20 min than 22 min. We have thus added this “attractor label value” to our model. However, this is a further question to be investigated, indicating just how complex the judgment of very long durations in humans is.

In sum, within the framework of internal clock models, we assume that the explicit judgments of short and very long durations share a common mechanism based on the functioning of an internal clock-like system. However, the judgment of very long durations involves additional mechanisms related to executive control processes associated with the awareness of the passage of time, which depends on the awareness of the self-time experienced during the interval duration, as embedded in our emotions and our activities. Awareness of the passage of time would therefore bring about a multiplicative change in the estimation of long durations.

## Supporting information

S1 Fig Mean duration, standardized error, and standard deviation of estimates for the different interval durations in the 2/8 and the 8/32-min condition, with the standard error and the confidence interval. (XLSX)
